# A new type of half-metallic fully compensated ferrimagnet

**DOI:** 10.1038/s41598-022-14561-8

**Published:** 2022-06-23

**Authors:** S. Semboshi, R. Y. Umetsu, Y. Kawahito, H. Akai

**Affiliations:** 1 Institute for Materials Research, Tohoku University, 2-1-1 Katahira, Aoba-ku, Sendai, 980-8577 Japan; 2 Center for Spintronics Research Network, Tohoku University, 2-1-1 Katahira, Aoba-ku, Sendai, 980-8577 Japan; 3 Center for Science and Innovation in Spintronics, 2-1-1 Katahira, Aoba-ku, Sendai, 980-8577 Japan; 4 Research Institute for Value-Added-Information Generation, Japan Agency for Marine-Earth Science and Technology, 3173-25 Showa-machi, Kanazawa-ku, Yokohama, Kanagawa 236-0001 Japan; 5 Department of Applied Physics, Graduate School of Engineering, Osaka University, 2-1 Yamadaoka, Suita, Osaka 565-0871 Japan; 6 The Institute for Solid State Physics, The University of Tokyo, 5-1-5 Kashiwanoha, Kashiwa, Chiba 277-8581 Japan

**Keywords:** Magnetic properties and materials, Information storage, Spintronics, Electronic structure

## Abstract

Half-metallic fully compensated ferrimagnets (HM-FCFMs) constitute a special class of half-metals exhibiting zero magnetization at zero temperature. While there have been a number of theoretical studies predicting the existence of such materials over the last 25 years, very few of those have been synthesized and observed that they exhibit expected properties. Herein, we demonstrate that a NiAs-type hexagonal-structured (CrFe)S compound could serve as an HM-FCFM material. It has a half-metallic nature of 100% spin-polarised Fermi surfaces and yet zero magnetisation at the ground state. The magnetisation shows linear behaviour as a function of the magnetic field at temperatures below the compensation temperature (~ 190 K). In addition, it shows a high magnetic coercivity of 3.8 T at 300 K. These magnetic features contribute to a significant development in the application of HM-FCFMs for spintronics devices.

## Introduction

A 100% spin-polarised current is a key feature in advanced spintronic data storage devices^1^, which are essential for quantum information processing and probabilistic computing^2^. This can only be achieved using half-metals. These materials have 100% spin-polarized Fermi surfaces, and hence, behave as metals in one spin direction and as insulators/semiconductors in the other^3^. Some half-metals have been developed to date, including: Heusler alloys^4–6^; transition metal oxides such as rutile^7^, spinel^8^, and perovskite compounds^9^; and diluted magnetic semiconductors^10,11^. However, all these half-metals show ferromagnetism. The development of half-metals with different magnetism will enable us to develop extremely powerful spintronic devices.

One possibility, proposed by van Leuken and de Groot in 1995^12^, is half-metallic fully compensated ferrimagnets (HM-FCFMs), commonly called half-metallic antiferromagnets. Obviously, literal antiferromagnets must be non half-metallic because their spin rotational symmetry is incompatible with half-metallicity. In contrast, fully compensated ferrimagnets, where the electron spin moments of crystallographically inequivalent magnetic sublattices are aligned antiparallel to each other with exactly zero net magnetic moment at 0 K, behave magnetically like antiferromagnets^13,14^, and simultaneously can be half-metals. A significant feature of HM-FCFMs is that they themselves create no external magnetic field, thereby any magnetostatic energy. Thus, HM-FCFMs would be useful for obtaining a 100% spin-polarised current in an environment without a magnetic field, facilitating novel spintronic devices.

There has been intensive theoretical research for over 25 years on HM-FCFM materials and a number of possible HM-FCFM materials and their properties have been predicted^15–27^. However, most of them are not practical or can appear only in unstable crystal structures. Exceptions are the bulk Heusler alloy Mn_1.5_V_0.5_FeAl^28,29^ and Mn_2_V_0.5_Co_0.5_Al melt-spun ribbons^30^ with *L*2_1_-type crystal structures: they have been demonstrated to be *N*-type ferrimagnetism as defined by Néel^13,14^. The band structure calculations for Mn_1.5_V_0.5_FeAl suggested that it had a half-metallic electronic structure^29^.

Akai and collaborators^27,31^ predicted possible existence of pnictide and chalcogenide HM-FCFM materials with NiAs-type structures on the basis of first-principles electronic-structure calculations. They concluded that, for transition-metal magnetic systems to be HM-FCFMs, the total number of *d*-electrons per magnetic ion must be equal to five (incidentally, the above examples of Mn_1.5_V_0.5_FeAl and Mn_2_V_0.5_Co_0.5_Al satisfy this condition), i.e., the sum of number of (effective) *d*-electrons for a pair of magnetic ions that couple antiferromagnetically to each other must be ten. Accordingly, some pnictides and chalcogenides with hexagonal NiAs-type structures, such as (CrFe)S, (CoV)Sb, and Cr/Fe-doped ZnS, are predicted to be HM-FCFM materials; however, none of them have been successfully synthesised to date.

Among the predicted candidates, (CrFe)S is attractive because it is composed of common elements with high Clarke numbers. Additionally, the Cr–Fe–S phase diagram indicates that it could be obtained via a conventional metallurgical process. If (CrFe)S is evidenced to exhibit a beneficial HM-FCFM behaviour, it will have significant impact on its practical application toward the development of spintronic devices. In this study, we attempted to synthesise NiAs-type chalcogenide (CrFe)S and evaluated its magnetic properties and half-metallicity.

## Results

### Synthesis of (CrFe)S compound

Hexagonal NiAs-type (CrFe)S with an Fe/Cr atomic ratio of 1 could be a HM-FCFM material primarily because it satisfies the aforementioned condition for the total number of *d*-electrons per magnetic ion. The phase diagrams (see Fig. S1A–C) of the Cr–Fe–S ternary alloy system^32,33^ indicate that the single-phase (CrFe)S has a wide compositional range at 1223 K with a Cr (or Fe) content of 0–50 at.% and S content of 50–55 at.%. However, a stoichiometric (Cr_25_Fe_25_)S_50_ (at.%) compound is expected to decompose into three phases, i.e., Fe-rich (CrFe)S, Cr-rich (CrFe)S, and α-Fe below 973 K, or three phases, i.e., Fe-rich (CrFe)S, (Cr_,_Fe)_3_S_4_, and α-Fe below 873 K. Our first attempt to synthesise a stoichiometric (Cr_25_Fe_25_)S_50_ (at.%) specimen by a powder sintering procedure, as reported by Sokolovich and Bayukov^32^, failed due to phase separation, viz. Fe-rich (CrFe)S and (Cr_,_Fe)_3_S_4_, as implied by the phase diagram. We then synthesised a slightly S-rich compound (Cr_23_Fe_23_)S_54_ at.% by sintering raw elemental powders to obtain single-phase (CrFe)S (see “Methods”).

### Structure of synthesized sample

**Figure 1:**
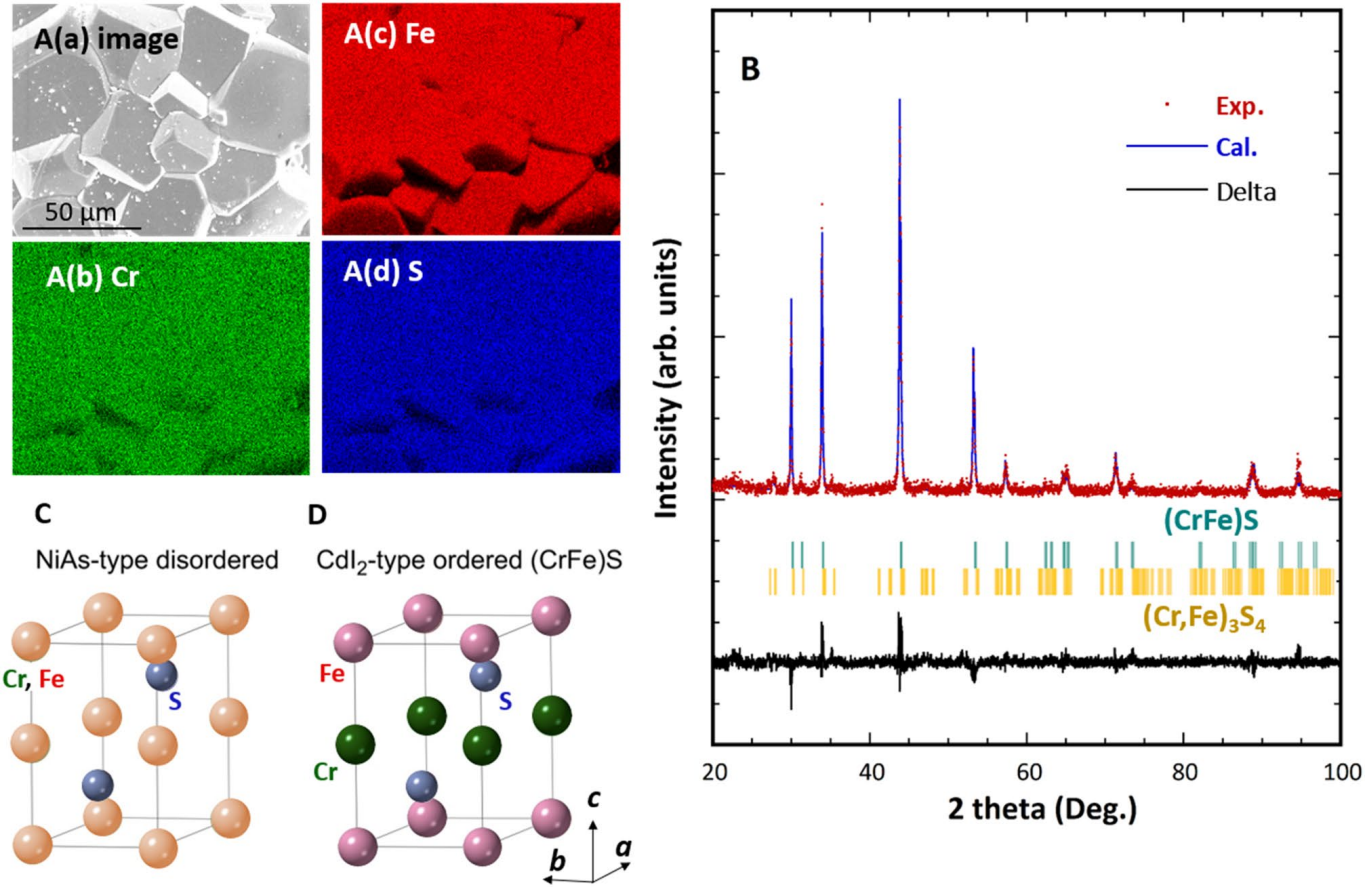
Structural properties. (**A**) Scanning electron microscopy–energy-dispersive X-ray spectroscopy images of the synthesised (CrFe)S sample: (A(a)) Backscattered electron image and elemental map for (A(b)) Cr, (A(c)) Fe, and (A(d)) S, taken from the same field of view as (A(a)). The synthesised compound comprised typical crystal grains with a size of ~ 40 μm. The constituent Cr, Fe, and S elements were homogeneously mixed; the dark contrast in (A(b–d)) was caused by surface irregularities. (**B**) XRD profile measured from the obtained specimen and one simulated by Rietveld analysis from NiAs-type disordered structure with an off-stoichiometric (Cr_21.3_Fe_21.3_*Vc*_7.4_)S_50_ composition, assuming that Cr atoms, Fe atoms, and 7.4 at.% of vacancies (*Vc*) are randomly distributed in the 2*a* sites are shown in the top. The peak positions of intermetallic compound (CrFe)S with a hexagonal structure and (Cr,Fe)_3_S_4_ with a monoclinic structure were indicated in the middle. The difference between the measured and simulated intensities is indicated at the bottom. The hexagonal (CrFe)S compound belongs to (**C**) NiAs-type disordered structure (space group *P*6_3_/*mmc* (No. 194)) or (**D**) CdI_2_-type ordered structure (space group *P*3*m*1 (No. 156)).

The chemical composition of the sample, as measured by inductively coupled plasma–atomic emission spectroscopy (ICP-AES), was 22.7Cr–23.3Fe–54.0S (at.%). The synthesised compound was composed of typical crystal grains in which the constituent elements were mixed homogeneously (see Fig. 1A).

The powder X-ray diffraction (XRD) pattern of the sample synthesised by sintering and quenching is shown in Fig. 1B. Main peaks were indexed primarily by the (CrFe)S compound with a hexagonal structure, as well as minor peaks indexed by the second phase of (Cr,Fe)_3_S_4_ with a monoclinic structure^34^. Rietveld analysis indicated the second phase of (Cr,Fe)_3_S_4_ compound was approximately as small as 4%. The primary phase of the (CrFe)S have NiAs-type structure (space group *P*6_3_/*mmc* (No. 194)), where the Cr and Fe atoms occupy the Wyckoff 2*a* positions (0, 0, 0) and the S atoms occupy the 2*c* sites (1/3, 2/3, 1/4), as shown in Fig. 1C. Here, Cr and Fe atoms are disordered, that is, they occupy the 2*a* sites randomly. If the Cr and Fe atoms form ordered structure, the specimen becomes a CdI_2_-type ordered lattice, which is one of the hexagonal NiAs-type (CrFe)S structures (Fig. 1D). The simulated XRD profiles of the CdI_2_-type ordered structure and NiAs-type disordered structure, which are shown in Fig. S1B,C (see Supplementary Information), were very similar and both of them well fit the experimental profile as shown in Figs. 1B and S1A. Thus, it was difficult to further clarify the crystal structure of the sample from the XRD profiles alone. Sokolovich and Bayukov^32^ concluded that their stoichiometric (CrFe)S compound, also synthesised by sintering and quenching, had a NiAs-type disordered structure, rather than a CdI_2_-type ordered structure, on the basis of observed Mössbauer spectra.

Since the sample synthesised in this study has a S-rich off-stoichiometric composition, the excess S atoms and/or vacancies (*Vc*) must be distributed in the 2*a* sites to retain a single-phase NiAs-type structure. The experimental diffraction peak intensities agreed well with the simulated ones for the (Cr_21.3_Fe_21.3_*Vc*_7.4_)S_50_ lattice as shown in Fig. S1E, rather than the simulated patterns for the (Cr_23_Fe_23_S_4_)S_50_ lattice as shown in Fig. S1D. This implies that the S-rich (CrFe)S compound synthesised in this study might have a NiAs-type disordered structure containing a certain number of vacancies in the 2*a* sites.

### Phase stability

**Figure 2:**
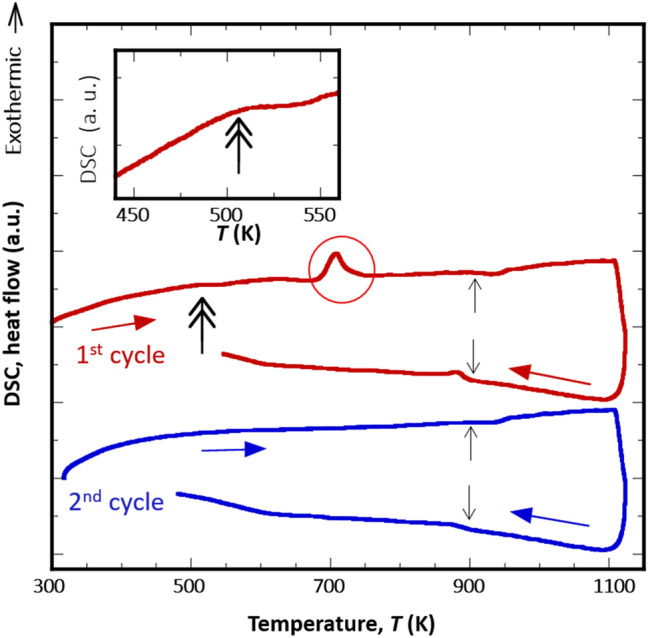
DSC results. Cyclic DSC heating and cooling curves for (CrFe)S synthesised by sintering and quenching, measured at the heating and cooling rates of 10 K/min. The exothermic peak at 700 K in the first heating curve (marked by solid circle) was attributed to the decomposition of the NiAs-type (CrFe)S phase. The single arrows at approximately 900 K and double arrow at 510 K (see the magnified curve in the inset) correspond to the recomposition of α-Fe and (Cr_,_Fe)_3_S_4_, and *T*_C_, respectively.

To examine the thermal stability of the (CrFe)S sample, cyclic differential scanning calorimetry (DSC) measurements were performed. During the first heating process, a relatively strong exothermic peak was observed at ~ 700 K (a solid circle in Fig. 2). This peak corresponds to the decomposition of the metastable (CrFe)S phase obtained by quenching to form three stable phases: Fe-rich (CrFe)S, α-Fe, and (Cr,Fe)_3_S_4_. We also observed reversible weak endothermic and exothermic peaks during heating and cooling processes, respectively, beginning at 900 K as marked by arrows in Fig. 2. This can be explained by the reconstitution of the three phases of Fe-lean (CrFe)S, α-Fe, and (Cr,Fe)_3_S_4_ into the single (CrFe)S phase above 900 K, and by decomposition of (CrFe)S into the three phases again, according to the phase diagram of the Cr–Fe–S ternary alloy system^32,33^. Therefore, quenching from a temperature higher than 900 K is required to obtain a single-phase of NiAs-type (CrFe)S compound with an Fe/Cr ratio of 1. The material is in the non-equilibrium phase state, but we can say from the DSC results that the phase is stable up to 700 K.

Figure 2 also shows a small step increment (inset of Fig. 2) at ~ 510 K (indicated by a double arrow) during the first heating. The step is associated with the magnetic transition of the (CrFe)S sample and the temperature should be the Curie temperature (*T*_C_), as will be again discussed in the next section. Since the phase was decomposed during the first heating process, no step was observed during the second heating process.

### Magnetic properties

**Figure 3:**
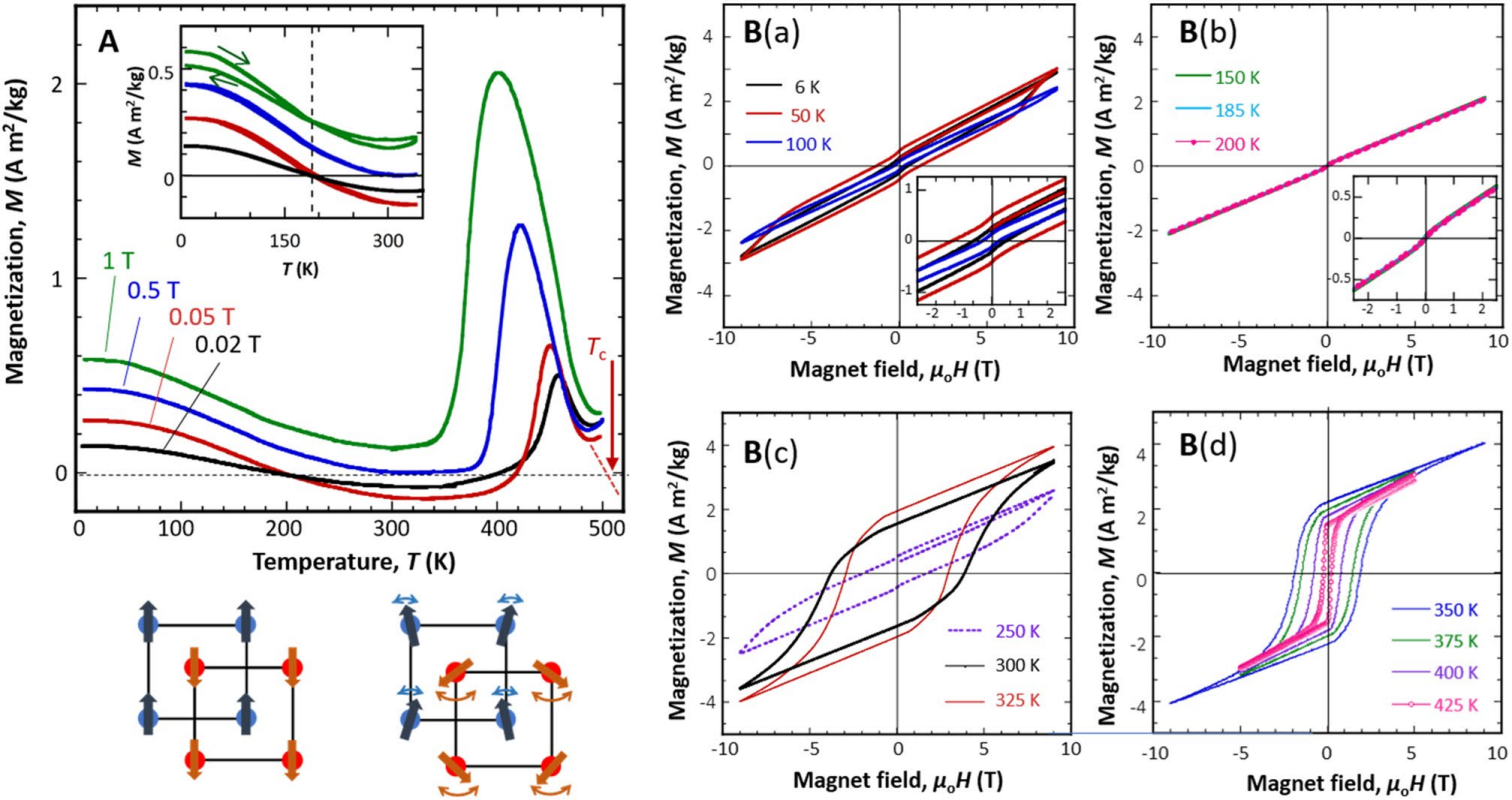
Magnetic properties. (**A**) Thermomagnetisation (*M*–*T*) curves of the (CrFe)S sample measured at 0.02 T, 0.05 T, 0.5 T, and 1 T in heating process (ZFC), together with magnetic field cooled (FC) *M-T* curves in the inset. Schematics of the magnetic state of the sublattices are illustrated in the bottom of (**A**). The magnetic moments in each sublattice were similar, but the strength of the exchange interaction depends on the magnetic-field and/or temperature. (**B**) Magnetisation (*M*–*H*) curves at different temperatures. (B(a)) 6–100 K: small hysteresis in the full loop; (B(b)) 150–200 K: perfectly linear relationship, indicating a fully compensated ferrimagnetic state; (B(c)) 250–325 K: abrupt hard-type magnetisation behaviour; and (B(d)) 350–425 K: magnetic coercivity (*H*_c_) gradually decreases with increasing temperature.

The thermomagnetisation (*M–T*) curves are shown in Fig. 3A. Under a low magnetic field (0.02 and 0.05 T), magnetisation decreased with increasing temperature, until the curve crossed zero around 190 K (i.e., the compensation temperature, *T*_comp_). This was followed by a convex downward behaviour in the higher-temperature region, with convex upward behaviour at temperatures just below *T*_C_. This behaviour is typical of *N*-type ferrimagnets^13,14^, except for the second zero and convex upward behaviour just below *T*_C_. Further, *T*_C_ was around 510 K, as indicated by the arrow in Fig. 3A, which well corresponds to the value obtained by DSC measurement (Fig. 2). Under a higher magnetic field (0.5 and 1 T), the magnetisation curve did not cross zero any longer, and the upward convex behaviour observed below *T*_C_ became more prominent. This suggests that magnetisation behaviour varies from *N*-type to *P*-type ferrimagnets as the magnetic field increases. The temperature dependence of the magnetisation is attributed to the presence of two magnetic sublattices in ferrimagnetic materials. Because the exchange interaction in each sublattice has different dependence on the magnetic field and temperature in general, the *M–T* curves may vary with the strength of the applied magnetic field^13,14^. See the inset of Figs. 3A and S2 (in Supplementary Information). The Inset of Fig. 3A indicates the *M*-*T* curves of heating process (ZFC) and magnetic field cooled (FC) ones. A small crossing at *T*_comp_ is observed in *M*-*T* measured at 1 T, showing that a slight magnetic field cooling effect exists.

The magnetisation (*M–H*) curves at temperatures ranging from 6 to 425 K are shown in Fig. 3B. Relatively low magnetisation (< 3 A m^2^/kg = 0.09 *μ*_B_ /f.u.) was observed for all *M–H* curves, even under a high magnetic field of 9 T. Further, the shape of the *M*–*H* curves was temperature dependent. At low temperatures (6*–*100 K, Fig. 3B(a)), the curves were nearly linear, with little hysteresis. At 6 K, the *M–H* curve deflects at approximately ± 7 T. This may suggest a slight change in the magnetic structure induced by the magnetic field, which has not yet been clarified. Over the temperature range from 150 to 200 K (Fig. 3B(b)), the *M–H* curves were linear, characteristic of fully compensated ferrimagnets^13,14^. At 250*–*325 K (Fig. 3B(c)), hard ferromagnetic behaviour suddenly appeared in the *M–H* curves. The magnetic coercivity (*H*_c_) had a maximum value of 3.8 T at 300 K. The high *H*_c_ value of 3.8 T at 300 K for the sample, which contains neither a rare-earth nor a noble-metal element, is noteworthy. This value is comparable with those of *L*1_0_-Mn_1.5_ Ga epitaxial films at room temperature^35^ and *ε*-Rh_*x*_Fe_2-*x*_O_3_ nanoparticles at 200 K (4.3 and 4.2 T, respectively)^36^. The high magnetocrystalline anisotropy of *L*1_0_-MnGa and ε-Fe_2_O_3_ was revealed by theoretical calculations^37,38^. However, the origin of the high magnetic anisotropy of the present system is not clear. The energy product, (*BH*)_max_, is relatively small because magnetisation is low owing to the ferrimagnetic properties of the material. Such a high magnetic coercivity above *T*_comp_ facilitates the application of this material for spintronic devices.

In the XRD patterns, the existence of a small amount of secondary phase of (Cr,Fe)_3_S_4_ is confirmed, as shown in Fig. 1B^34^. Therefore, the effects due to the secondary phase on the magnetic properties of matrix should be discussed. The magnetic properties of Cr_2_FeS_4_ with spinel-type cubic structure, showing ferrimagnetism with the Curie temperature of 170 K and a blocking temperature of 60 K^39,40^, have been intensively investigated because of an interest in the semiconducting properties^39,41^. However, our Rietveld analysis indicated that the observed diffraction peaks relating to the secondary phase could not be indexed as a spinel-type cubic structure but as a monoclinic structure. The magnetic properties of the monoclinic Cr_2_FeS_4_ phase were investigated by a neutron diffraction study^42^ and Mössbauer spectroscopy^43^. The results of those investigations showed that the monoclinic (Cr,Fe)_3_S_4_ was a weak antiferromagnet. Thus, we conclude that the effects of the magnetic component of a small amount of secondary phase on the magnetism (CrFe)S matrix phase is negligibly small.

### Half-metallicity

**Figure 4:**
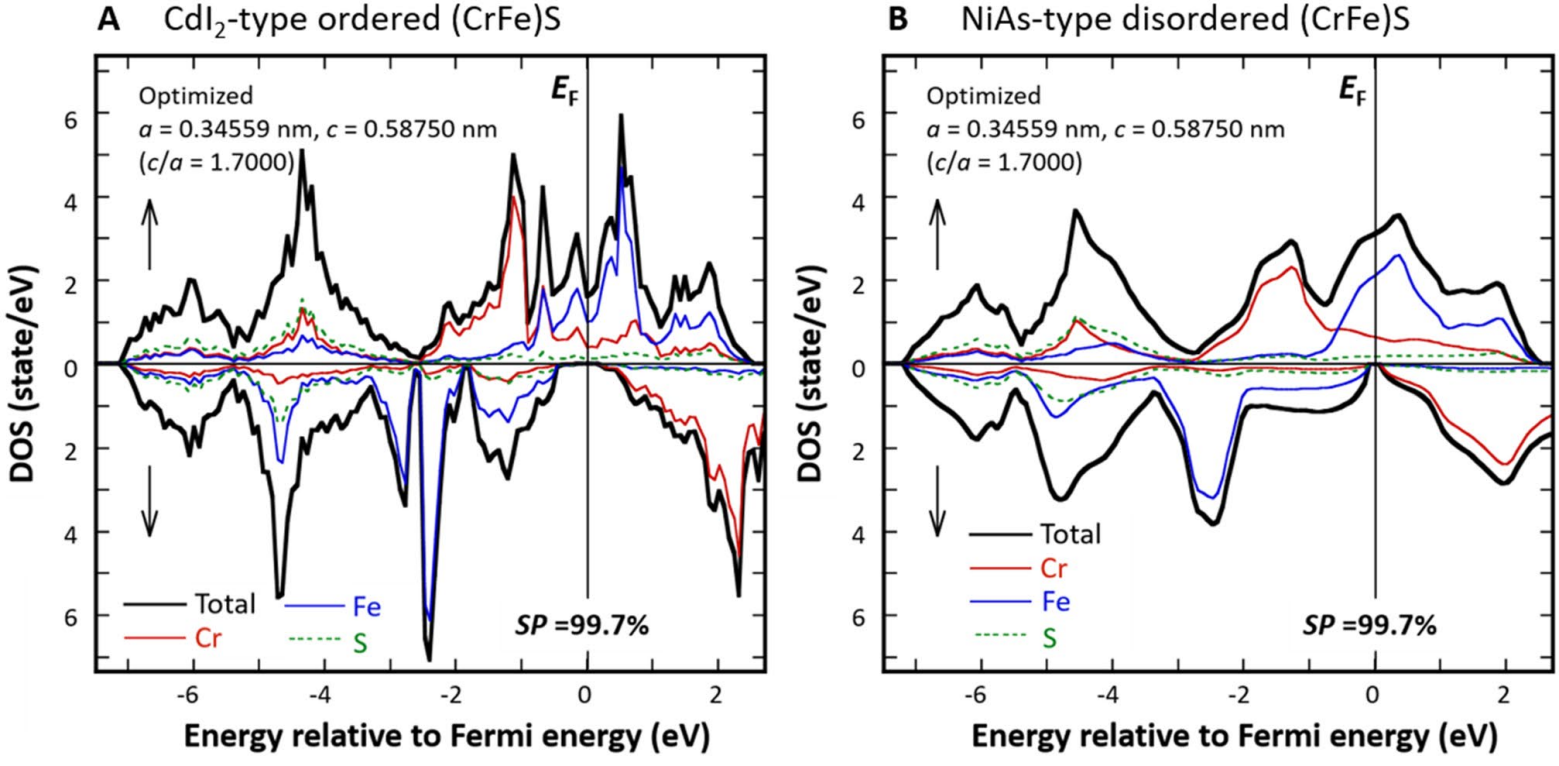
Density of states (DOS). Partial DOS of Cr, Fe, and S and the total DOS of (**A**) CdI_2_-type ordered and (**B**) NiAs-type disordered (CrFe)S with a stoichiometric composition, calculated using the optimised lattice parameters by the KKR method. The coherent potential approximation (CPA) method was used to calculate the disordered state. Partial DOS refers to the angular-momentum-decomposed DOS within the Voronoi cell surrounding each constituent atom. *SP*: spin polarization.

The synthesised (CrFe)S samples are expected to exhibit fully compensated ferrimagnetic properties and half-metallicity simultaneously. To examine the half-metallicity of this material, we performed electronic structure calculations of the stoichiometric CdI_2_-type ordered compound and NiAs-type disordered (CrFe)S compound using the first-principles full-potential KKR-CPA method: the calculated densities of states (DOS) are shown in Fig. 4A,B, respectively (see “Methods”). The partial DOS refers to angular-momentum-decomposed DOSs within each Voronoi cell surrounding a constituent atom. For the stoichiometric ordered or disordered (CrFe)S, the Fermi level (*E*_F_) is located in a band gap (or a deep valley) for the spin-down state, while it is located within the band for the spin-up state, indicating that the (CrFe)S system is a half-metal. Further, the total magnetic moment of the system is zero within numerical accuracy, which proves the fully compensated ferrimagnetism of the systems.

The lattice parameters of stoichiometric NiAs-type (CrFe)S were predicted to be *a* = 0.3456 nm and *c* = 0.5875 nm from first-principles calculations. However, the experimental lattice parameters obtained from the XRD profile shown in Fig. 1B were *a* = 0.3446 nm and *c* = 0.5743 nm, with an expected experimental accuracy of ± 0.0010 nm. The first-principles calculations of the stoichiometric CdI_2_-type ordered and NiAs-type disordered (CrFe)S compounds using the experimental lattice parameters gave DOS (see Fig. S3 in Supplementary Information) and physical properties (e.g. magnetic moment and Curie temperature) that were slightly different from those obtained using the theoretical lattice constants, as listed in Table 1. However, there was no essential difference between the results obtained with two different sets of lattice constants.

**Table 1:** Physical properties of CdI_2_-type ordered and NiAs-type disordered (CrFe)S compound.

Structure (space group)	Atom	Site Wyckoff position	Magnetic moment (*μ*_B_)	*T*_C_ (K)	Ordering energy (eV)	Spin polarization (%)
CdI_2_-type ordered (No. 156)	Fe	1*a* (0, 0, 0)	− 2.94 (− 2.86)	1224 (1259)	–	99.7 (99.7)
	Cr	1*a* (0, 0, 1/4)	3.12 (3.03)			
	S	1*b* (1/3, 2/3, 1/4) 1*c* (2/3, 1/3, 1/4)	− 0.10 (− 0.09)			
NiAs-type disordered (No. 194)	Fe	2*a* (0, 0, 0)	− 3.06 (− 2.98)	1026 (1149)	0.325 (0.356)	99.7 (96.9)
	Cr	2*a* (0, 0, 0)	3.27 (3.16)			
	S	2*c* (1/3, 2/3, 1/4)	0.11 (− 0.10)			

The magnetic moment, the Curie temperature (*T*_C_), ordering energy (i.e., the difference in total energy between the CdI_2_-type ordered structure and NiAs-type disordered structure), and spin polarization were calculated by the KKR method.

As was mentioned, the (CrFe)S sample synthesised in this study has an off-stoichiometric composition. We assume that the 2*a* sites in the NiAs-type structure are randomly occupied by Cr, Fe, excessive S atoms, and *Vc*. For example, if assumed that the structure is represented as (Cr_21.8_Fe_21.8_S_1.4_*Vc*_5.0_)S_50_, the DOS is essentially the same as that of stoichiometric NiAs-type (CrFe)S (Figs. S3 and S4 in Supplementary Information). Thus, it is concluded that the half-metal-type electronic state is retained when the total number of *d*-electrons per magnetic ion is five (when the Fe/Cr ratio of (CrFe)S is 1 in the case of NiAs-type (CrFe)S) even if the lattice parameters fluctuate slightly and/or the S content deviates from the stoichiometric composition (i.e., an introduction of a slight amount of vacancy at 2*a* sites). Based on this conclusion, it is expected that (CrFe)S compounds with S contents of 50–55 at.%, including the (Cr_23_Fe_23_)S_54_ compound synthesised in this study, should show HM-FCFM behaviour.

### Other calculated electronic and magnetic properties

The physical properties of the CdI_2_-type ordered and NiAs-type disordered (CrFe)S systems, which were calculated using the optimised or experimental lattice constants, are listed in Table 1. In the NiAs-type disordered (CrFe)S system with optimised lattice constants, the spin magnetic moments of Cr and Fe are 3.27 and − 3.06 *µ*_B_, respectively. Since the electronic states of both Cr and Fe atoms are rather localised, they carry relatively large magnetic moments. The magnitudes of the magnetic moments are similar and allow the existence of fully compensated ferromagnetism even in a disordered state. The total energy of the CdI_2_-type ordered structure is ~ 300 meV lower than that of the NiAs-type disordered structure, suggesting that the former structure is more stable at the ground state. However, in the experiments described herein, the sample was obtained by quenching from 1253 K to obtain a single-phase (CrFe)S compound, avoiding phase separation. In this situation, obtaining a disordered state should not be surprising because the difference in the total energies between the ordered and disordered states is relatively small.

The *T*_C_ value was calculated within the mean field approximation using the exchange interaction, *J*’s , that were calculated by Liechtenstein’s method^44^. It is generally known that *T*_C_ calculated in this way is considerably higher than the corresponding experimental value. Under otherwise the same numerical setup, the *T*_C_ of the NiAs-type disordered compound was ~ 200 K lower than that of the CdI_2_-type ordered compound. The spin polarization of the CdI_2_-type ordered compound was calculated to be 99.7% (not 100% due to an artifact mentioned later), indicating that the system is a half-metal. Furthermore, the property is robust against the disorder and changes in the lattice constants. This would be a significant advantage for various applications.

### Potential applications

**Figure 5:**
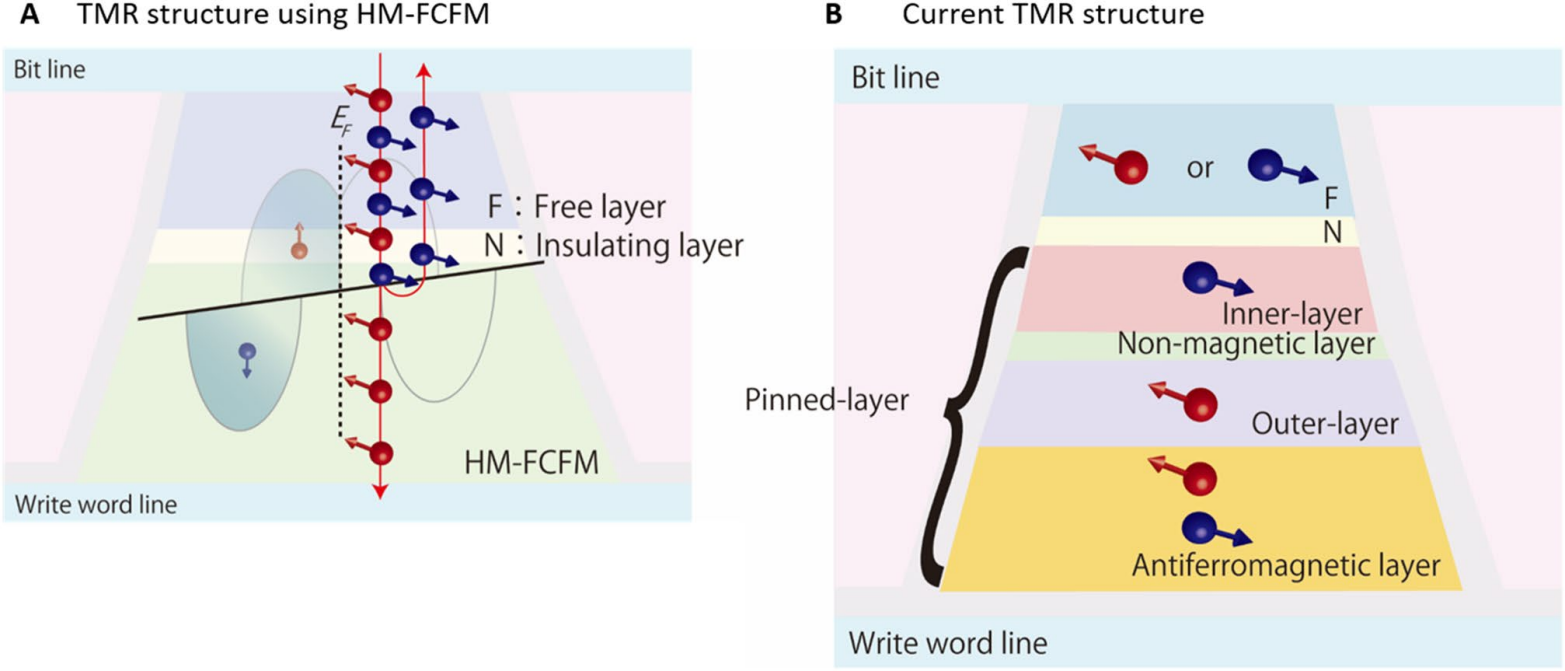
Illustration of a TMR device. (**A**) Schematic of a potential TMR device with a ferromagnetic free layer (F), non-magnetic (insulator) layer (N), and pinned layer of half-metallic fully compensated ferrimagnet (HM-FCFM). The HM-FCFM layer acts as an electron conductor in one spin orientation (up-spin, shown in red) but as an insulator in the opposite orientation (down-spin, shown in blue), as depicted in the schematic DOS for antiferromagnetic coupling. (**B**) Conventional TMR structure with a pinned layer, comprising an inner layer, a non-magnetic layer (e.g., Ru-layer), an outer layer, and an antiferromagnetic layer. The device in (**A**), in which the HM-FCFM layer replaces the pinned layer in (**B**), facilitates a higher TMR ratio and lower leakage current than the device in (**B**).

Ideally, in its ground state, NiAs-type (CrFe)S acts as an electron conductor with one spin orientation (e.g. spin-up state, shown in red in Fig. 5A) but as an insulator with the opposite spin orientation (spin-down sate, shown in blue in Fig. 5A), which leads to the generation of current with high spin-polarization. This feature has attracted interest because it facilitates potential applications in the spintronics field. For example, compared to conventional tunnelling magneto resistors (TMRs), which contain ferromagnetic (e.g., permalloy) inner and outer layers (Fig. 5B), TMR devices containing a HM-FCFM layer should have drastically improved TMR ratios^45,46^, as the leakage current would be minimised as generally expected for devices using half-metals. The Julliére model suggests that the TMR ratio in magnetic tunnelling junctions is infinite when complete half-metallic ferromagnets are used as the electrode layers^47^. More important advantage of using HM-FCFM for TMR is that it enables us to get rid off all complicated structures that are necessary to fix the direction of magnetization of a pined layer (Fig. 5B). Such elaborations are necessary because a ferromagnetic pinned-layer has magnetization and responds to an external field: for the TMR to work properly the pined layer should not respond to external fields. The HM-FCFM automatically solves this problem because it carries no magnetization, and hence, would not respond to any external fields. This fact, together with another important fact that it does not cause any stray fields, greatly simplifies the construction of TMR elements and also contributes to improve massive integrations. Additionally, it is noteworthy that (CrFe)S is composed of common elements with high Clarke numbers. Therefore, the innovation that might accelerate a major development in spintronics devices is expected by using HM-FCFMs as possible core materials.

## Discussion and summary

In this study, we confirmed that the fully compensated ferrimagnetism is observed when the Fe/Cr ratio in the (CrFe)S compound is 1. If combined this fact with the results obtained by first-principles calculation, we can conclude that the systems are very likely to be half-metallic compensated ferromagnets, The calculations confirm that the half-metallicity is rather robust; the property is retained even if the lattice parameters fluctuate slightly and the S content deviates from the stoichiometric composition, suggesting that the S-rich off-stoichiometric (CrFe)S sample synthesised in this study is a half-metallic. Since the (CrFe)S system is half-metallic, the properties of fully compensated ferrimagnetism are also preserved. The deviation of spin polarization from 100%, as shown in Fig. 4, is due to a non-zero imaginary part attached to the Fermi level, which is necessary for the numerical calculations using KKR-CPA.

Despite attempts for over 25 years to develop HM-FCFM materials, only the Mn_1.5_V_0.5_FeAl bulk Heusler alloy has been reported as a potential candidate thus far^28,29^. Here, we successfully synthesised a NiAs-type (CrFe)S compound and confirmed its behaviour as a new type of HM-FCFM material. The HM-FCFM (CrFe)S material was developed on the basis of the *d*-electron number rule proposed previously^27,31^. Note that the Mn_1.5_V_0.5_FeAl bulk Heusler alloy also satisfies the *d*-electron number requirement, as mentioned already. Thus, the *d*-electron number rule should be a principal guideline for fabricating new HM-FCFM families in the future. We are confident that the findings of this study will trigger new innovation in the spintronics field.

## Methods

### Synthesis of off-stoichiometric (CrFe)S compound

Off-stoichiometric (CrFe)S with a nominal composition of (Cr_23_Fe_23_)S_54_ was synthesised by a powder-metallurgy process. Raw elemental powders of pure Fe (> 99.9 wt% purity, particle size < 53 μm), Cr (99.9 wt%, 180–300 μm), and S (99.99 wt%, < 75 μm) were weighed to achieve the nominal composition and mixed well using a rotating mixer to obtain a homogeneous mixed powder. This powder was packed into a die made of high-speed steel, and then compressed by cold uniaxial pressing under a pressure of > 300 MPa at room temperature (293 K). Following this procedure, since the Cr and Fe powder were deformed plastically, a high-density cylindrical compact was obtained with a 10 mm diameter and a 10 mm height. The green compact was encapsulated in a quartz tube under an Ar atmosphere (99.99999% Ar) with a pressure of 0.1 MPa and then sintered at 1253 K for 24 h to be fully homogenised (according to the Cr–Fe–S phase diagram, a sintering temperature of > 1223 K is required). Then, the sample was quenched in water. The obtained sample was gently crushed into a coarse powder using an agate mortar.

### Experimental characterization

The appearance of the sintered sample was examined by scanning electron microscopy (SEM; Hitachi S-3400N) with a conventional tungsten emitter, while its compositional homogeneity was evaluated using a coupled energy-dispersive X-ray spectroscopy (EDS) system at an accelerating voltage of 15 kV. The chemical properties of the sample were evaluated by ICP-AES using a Thermo Fischer Scientific IRIS Advantage DUO instrument. The phase and structure of the sample were identified by powder XRD measurements (Rigaku Rint-Ultima III) at room temperature with Cu Kα radiation at 40 kV. The diffraction angle (2θ) range was 20–100° with a step size of 0.02°. Rietveld analyses were carried out using “*Z-Rietveld 1.1.3*” soft wear^48,49^. The phase stability of the sample was examined by DSC (SII EXSTAR 6000) from room temperature (293 K) to 1100 K at heating and cooling rates of 10 K/min under a flow of 99.99999% Ar (50 mL/min).

Magnetic measurements were performed at a heating rate of 2 K/min using a superconducting quantum interference device magnetometer and a vibration sample magnetometer equipped with a physical property measurement system (Quantum Design Ltd).

### Theoretical calculation of the electronic structure

First-principles calculations were performed using the density functional theory framework, with the local density approximation/generalized gradient approximation (LDA/GGA), using the all-electron full-potential Korringa–Kohn–Rostoker Green’s function method (FPKKR)^50^ combined with the coherent potential approximation (CPA)^51,52^. The CPA procedure is required to simulate realistic situations in which the S atoms occupy some of the transition metal sites (2*a* sites); therefore, the system is no longer stoichiometric. We used the GGA scheme proposed by Perdew, Burke, and Ernzerhof (PBE)^53^; however, the results were not significantly dependent on the choice of the LDA/GGA schemes. We assumed a CdI_2_-type ordered structure for the systems where a Cr atom occupies one of the two transition metal sites in the NiAs-type structure, while an Fe atom occupies the other transition metal site in each unit cell, as observed in Fig. 1D. We also considered the situation in which the Cr and Fe atoms occupy two transition metal sites randomly, with a probability of occupation of 0.5 (this is the case for the NiAs-type structure presented in Fig. 1C). In such cases, we applied the FPKKR-CPA to consider the random arrangements. The same procedure was used when the partial substitutions of the Cr and Fe atoms for S atoms and vacancies were considered.

The Curie temperatures of the systems were calculated by constructing a low-energy effective Hamiltonian using Liechtenstein’s formula^44^. Once the effective Hamiltonian was obtained, which takes the form of the Heisenberg model with a relatively long-range coupling constant *J*_*ij*_, the Curie temperature was calculated using the mean-field approximation. Although the mean-field approximation generally overestimates the Curie temperature, this is not particularly significant for the present system owing to the highly concentrated magnetic ions.

## Supplementary Information

Supplementary Information.

## Data Availability

The datasets generated during and/or analysed during the current study are available from the corresponding author on reasonable request.
