# Supplementary material for: A new type of half-metallic fully compensated ferrimagnet
Source: Sci Rep. 2022 Jun 23;12:10687. doi: 10.1038/s41598-022-14561-8 (PMC9226010; doi:10.1038/s41598-022-14561-8)
Supplement: Supplementary file 1 — Supplementary Information. [file 41598_2022_14561_MOESM1_ESM.pdf]

Supplementary Information for

A New Type of Half-Metallic Fully Compensated Ferrimagnet

S. Semboshi, R.Y. Umetsu, Y. Kawahito, H. Akai*.

*Corresponding author email: akai@cp.prec.eng.osaka-u.ac.jp ORCID: 0000-0002-3551-7443

This PDF file includes

Supplementary Text

Figs. S1–S5

Supplementary Text

Phase diagrams

The isothermal phase diagrams of Cr-Fe-S ternary system at 873 to 1223 K were reported as shown in **Fig. S1** (*1*). The single-phase (CrFe)S has a wide compositional range at 1223 K with Cr (or Fe) content ranging from 0 to 50 at.% and S content ranging from 50 to 55 at.%. However, the single-phase region narrows as the temperature decreases. The phase diagrams indicate that, to obtain the (CrFe)S compounds with the Fe/Cr ratio = 1.0, the alloy with a composition around $(Cr_{23}Fe_{23})S_{54}$ (at.%) must be heat-treated at a temperature higher than 973 K and then rapidly quenched.

Crystal structure

The powder XRD profile of the sample synthesized by sintering and quenching (Fig. S2A) showed that the sample had a hexagonal NiAs-type structure. To evaluate this result in detail, we simulated the XRD profiles based on possible NiAs-type (CrFe)S structures (Figs. S2(B-E)). Figure S2B shows the XRD profile corresponding to the stoichiometric CdI_2-type ordered structure illustrated in Fig. 1D, while Fig. S2C shows the stoichiometric NiAs-type disordered structure illustrated in Fig. 1C. There is no appreciable difference between the simulated XRD profiles of the CdI_2-type ordered structure and NiAs-type disordered structure. The experimental XRD profile shown in Fig. S2A mostly fit the simulated profiles for both the stoichiometric CdI_2-type ordered and NiAs-type disordered structures. This suggests that it is difficult to evaluate the degree of ordering from the XRD analysis alone. However, Sokolovich and Bayukov concluded from Mössbauer spectroscopy experiment that similar but stoichiometric (CrFe)S specimens synthesized by sintering and quenching have a NiAs-type disordered structure rather than a CdI_2-type ordered structure (*2*).

The chemical composition for the sample obtained by ICP-AES measurements was 22.7% Cr–23.3% Fe–54.0% S (at.%). This is nearly identical to the nominal composition of 23% Cr–23% Fe–54% S (at.%). Since the (CrFe)S sample synthesized in this study has a S-rich off-stoichiometric composition with an Fe/Cr ratio of approximately 1, the excess S atoms and/or vacancies (Vc) are expected to be distributed in the 2*a* sites to retain a single-phase NiAs-type structure. Figs. S2D and E present off-stoichiometric NiAs-type structures with the composition $(Cr_{23}Fe_{23})S_{54}$. Fig. S2D is obtained by assuming that the excess S atoms (4 at.%) randomly substitute for Cr/Fe at the 2*a* sites (i.e., the sample can be represented as $(Cr_{23}Fe_{23}S_4)S_{50}$), while Fig. S2E is the results assuming that 7.4 at.% of the 2*a* sites are randomly occupined by vacancies (V_C) (i.e., $(Cr_{21.3}Fe_{21.3}Vc_{7.4})S_{50}$, in which the ratio of Cr, Fe, and S atoms is 23:23:54). The experimental diffraction intensities for 010 and 011 in Fig. S2A agree

well with the simulated patterns for the $(Cr_{21.3}Fe_{21.3}Vc_{7.4})S_{50}$ lattice shown in Fig. S2E, rather than the simulated patterns for the $(Cr_{23}Fe_{23}S_4)S_{50}$ lattice shown in Fig. S2D. This indicates that the S-rich (CrFe)S compound synthesized in this study potentially has a NiAs-type disordered structure containing a certain number of vacancies in the 2*a* sites. It may be noted that 7.4% of vacancies in the 2*a* sites might be excessive for retaining the crystal structure and electronic state of the NiAs-type lattice. Therefore, it may be reasonably assumed that the 2*a* sites in the experimental samples are occupied not only by the vacancies, but also by a portion of excess S atoms along with Cr and Fe atoms. To elucidate the detailed structure and degree of ordering of the (CrFe)S compound synthesized, neutron diffraction analysis is ultimately required, which will be reported in the future.

Temperature dependence of the magnetization

Behavior of the *M-T* curevs of ferrimagnets differs depending on the strength of the exchange interaction of the sublattices, as predicted by Néel *(3,4),* and categorised into several types. Fig. S3 indicates the examples of the temperature dependences of the magnetization of each sub-lattice (red and blue) and the total magnetization (green), which is simply the sum of the magnetizations of the two sub-lattices. The R, N, and P-type behaviours have been already predicted by Néel. The right-bottom figure shows the possibility of the upward convex beahviour observed just below the Curie temperature (called here as NP-type), in which the temperature dependence of magnetization of each sub-lattice varies from N to P-type. Thus, the upward convex behavior can be seen as a result of the mixture of different behaviours in the sublattice magnetizations.

Theoretical calculation of the density of states

It is important for first-principles calculations to use proper crystallographic parameters such as lattice parameters and atomic arrangement in the unit cell. The lattice parameters of stoichiometric NiAs-type (CrFe)S are predicted to be $a = 0.3456$ nm and $c = 0.5875$ nm based on first-principles calculations. The DOS is also drawn, as shown in Fig. 4. The lattice parameters of the (CrFe)S sample in this study, however, were measured to be $a = 0.3446$ nm and $c = 0.5743$ nm based on the XRD profile shown in Fig. S2A with an expected experimental accuracy of ±0.0010 nm. If we performed the first-principles calculations using the experimental lattice parameters, we obtained the DOS shown in Fig. S4 for the stoichiometric CdI_2-type ordered and NiAs-type disordered (CrFe)S compounds, as well as the values of physical properties such as magnetic moment and Curie temperature. We find that these are not significantly different from the theoretical values obtained by first-principles calculations, presented in Fig. 3 and Table 1. This indicates that the (CrFe)S compounds exhibits a half-metallicity even if the lattice parameters fluctuate slightly.

Since the (CrFe)S sample synthesized in this study has an off-stoichiometric composition, the atomic arrangement cannot be strictly determined from the XRD profile as discussed before. We assume that the 2*a* sites in the NiAs-type structure are randomly occupied by Cr, Fe, excessive S atoms, and vacancies (*Vc*). For example, if the structure assumed represented as $(Cr_{21.8}Fe_{21.8}S_{1.4}Vc_{5.0})S_{50}$, the DOS becomes one shown in Fig. S5. We can see that the DOS shown in Fig. S5 is essentially the same as the DOS shown in Figs. 5 and S4. Thus, it is concluded that the half-metal-type electronic state is retained as long as the total number of *d*-electrons per magnetic ion is 5 (when the Fe/Cr ratio of the (CrFe)S compound is 1 in the case of the NiAs-type (CrFe)S compound), even if some vacancies exist and the S content deviates from the stoichiometry. We thus conclude that (CrFe)S compounds with S content ranging from 50 to 55 at.%, including the $(Cr_{23}Fe_{23})S_{54}$ compound synthesized in this study exhibit half-metallic fully compensated ferrimagnetism.

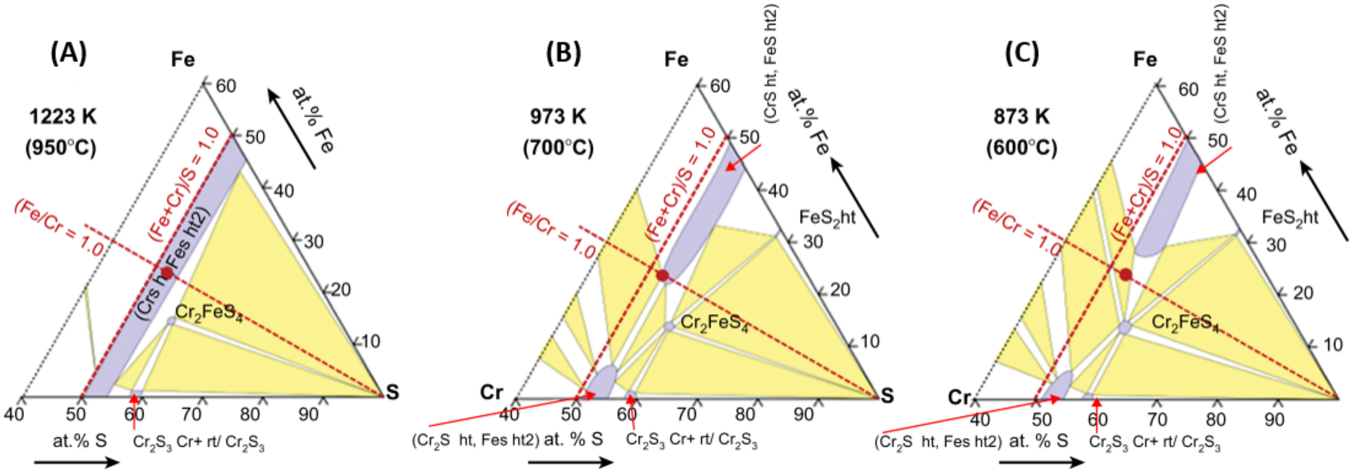

Fig. S1. Partial phase diagrams of the Cr–Fe–S ternary system at (A) 1223 K, (B) 973 K, and (C) 873 K (*1*). The (CrFe)S with the composition $(Cr_{23}Fe_{23})S_{54}$ systhesized in this study are marked by the red circles. The red dotted lines indicate the compositional ratios of Fe/Cr = 1.0 and (Fe+Cr)/S = 1.0.

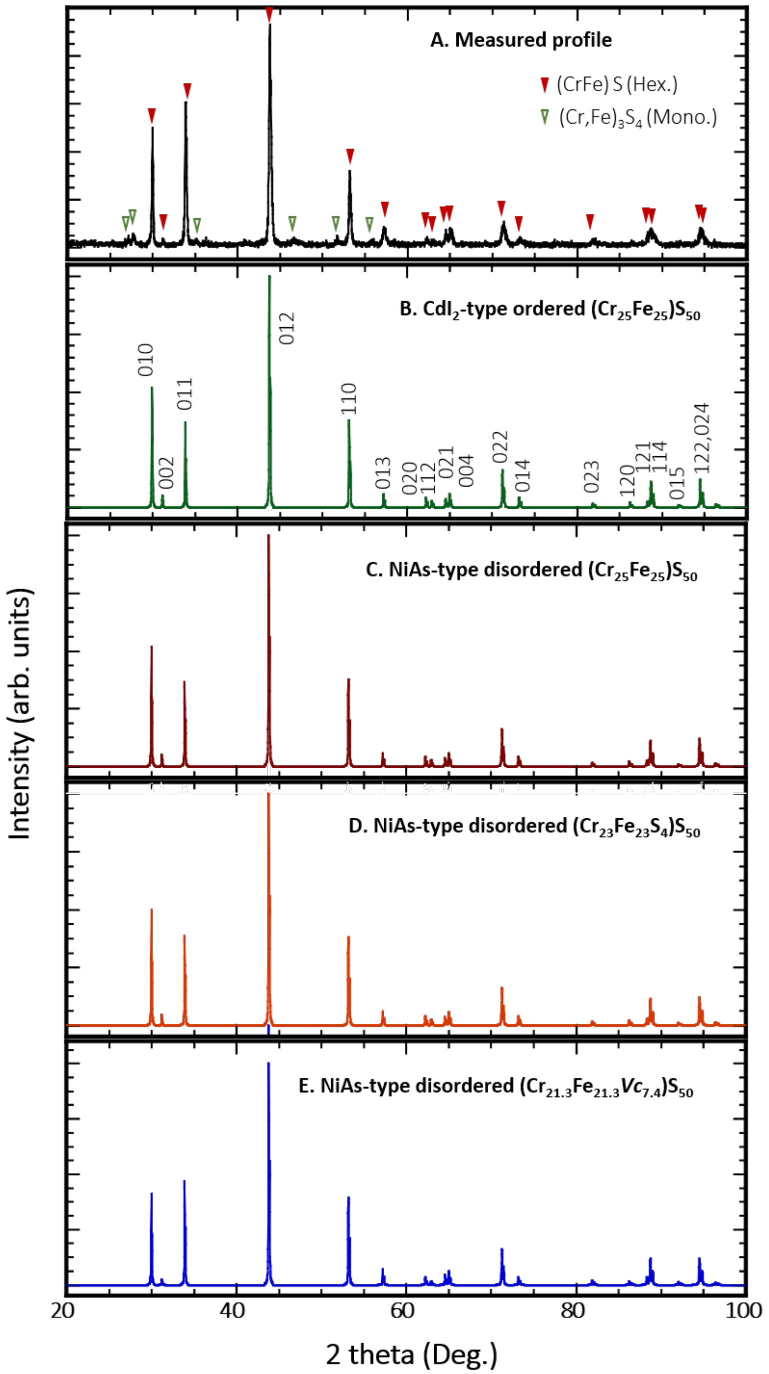

Fig. S2. **(A)** Experimental X-ray diffraction profile of specimen synthesized in this study, and **(B–E)** simulated profiles for **(B)** CdI_2-type ordered structure with stoichiometric composition, with Cr (25 at.%) and Fe (25 at.%) ordered in the 2*a* (0, 0, 0) sites and S (50 at.%) in the 2*c* sites for the Wyckoff position (shown in Fig. 1D); **(C)** NiAs-type disordered structure with stoichiometric composition (Fig. 1C); **(D)** NiAs-type disordered structure with an off-stoichiometric $(Cr_{23}Fe_{23}S_4)S_{50}$ (at.%) composition, assuming that Cr, Fe, and excess S atoms are randomly distributed in the 2*a* sites; and **(E)** NiAs-type disordered structure with an off-stoichiometric $(Cr_{21.3}Fe_{21.3}Vc_{7.4})S_{50}$ composition, assuming that Cr atoms, Fe atoms and 7.4 at.% of vacancies (*Vc*) are randomly distributed in the 2*a* sites.

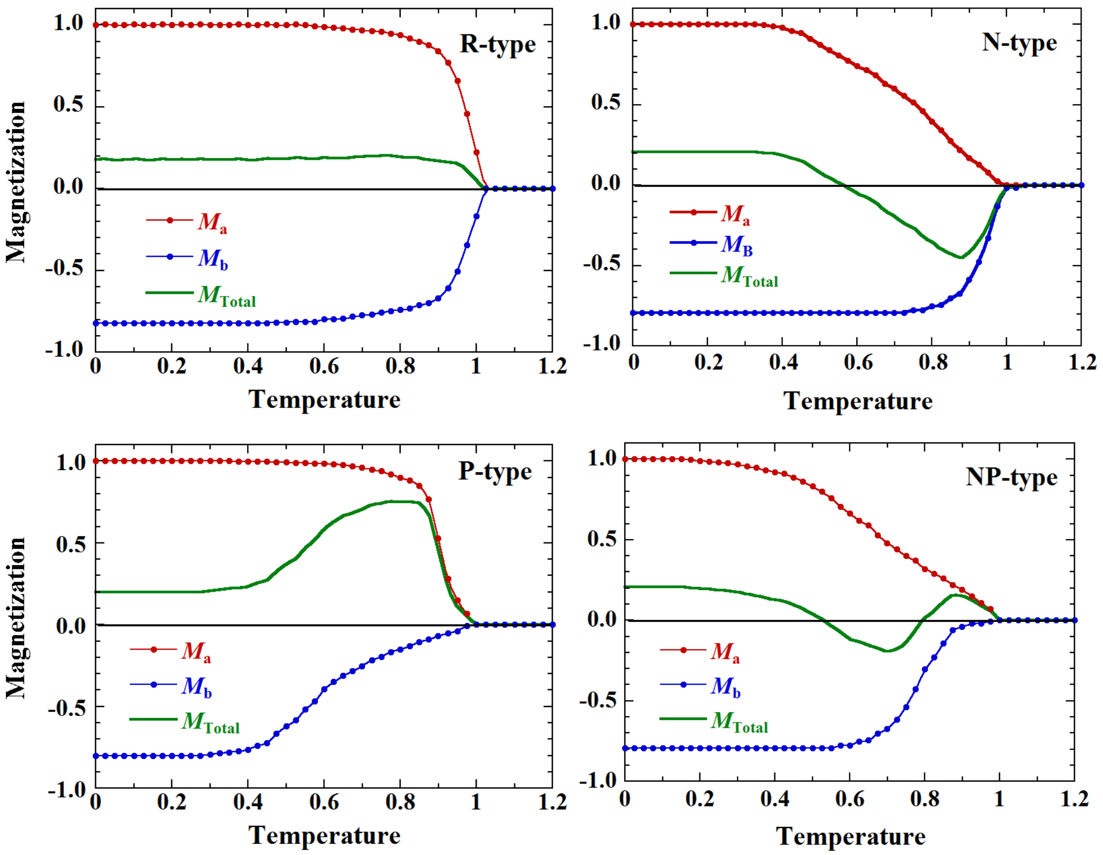

Fig. S3. Examples of the temperature dependencies of the magnetization of each sub-lattice (red and blue) and the total magnetization (green) that is simply obtained as a sum of the magnetizations of the two sub-lattices. R, N, and P-type behavior have been predicted by Néel *(2,3)*. The right-bottom figure (NP-type) indicates a possibility of the upward convex beahviour, observed experimentally, just below the Curie temperature, where the temperature dependence of magnetization of each sub-lattice varies from N to P-type.

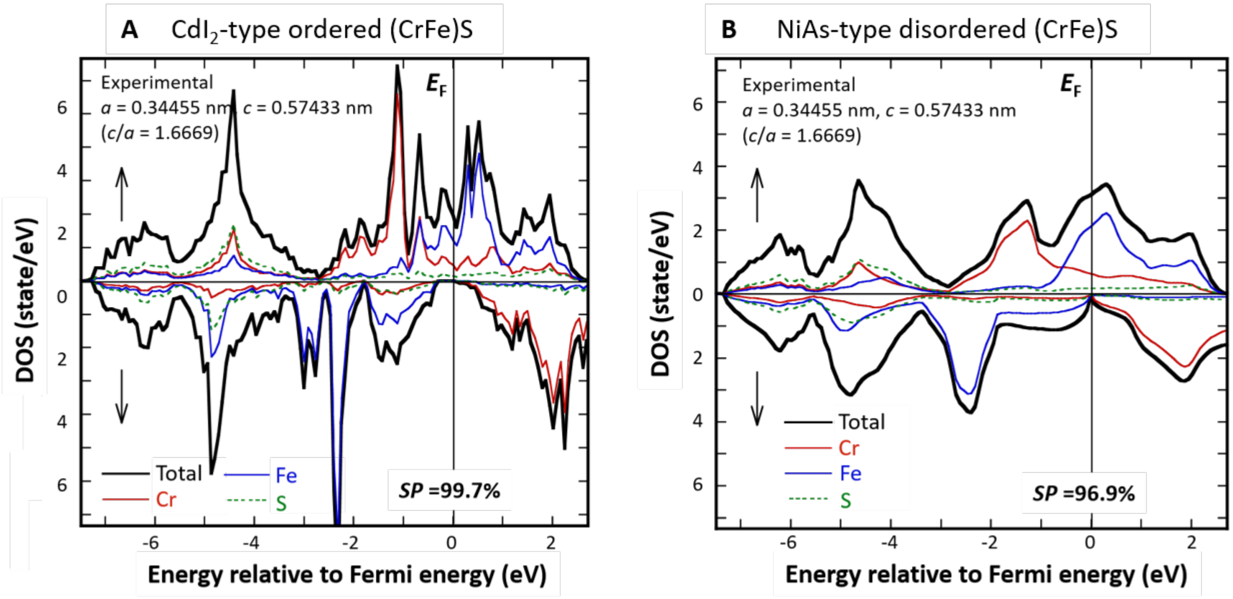

Fig. S4. Partial DOSs of Cr, Fe, and S atoms and the total DOSs of (A) CdI_2-type ordered and (B) NiAs-type disordered (CrFe)S (both in stoichiometric composition), calculated using the lattice parameters obtained from the experimental XRD pattern shown in Fig. S2A. The disordered states were calculated using the coherent potential approximation (CPA).

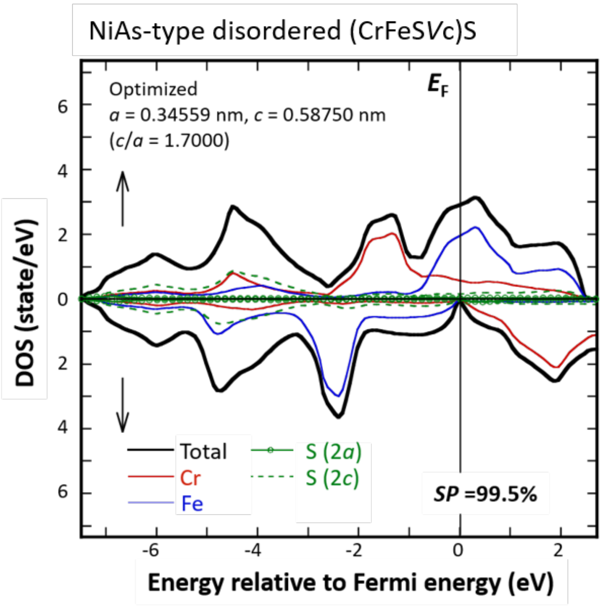

Fig. S5. Partial DOSs of Cr, Fe, and S and the total DOS of an off-stoichiometric NiAs-type disordered $(Cr_{21.8}Fe_{21.8}S_{1.4}Vc_{5.0})S_{50}$. The lattice parameters are obtained by the optimized calculation. The vacancies are introduced in the 2*a* site, The structure is close to the structural model concluded from the experimental XRD pattern. The spin polarization is kept almost perfect despite the content of S becomes to be slightly rich.

Suppremental references

1. ASM Alloy phase diagram database, https://matdata.asminternational.org/apd/index.aspx.
2. Sokolovich, V. V. & Bayukov, O. A. Mossbauer spectra of $Fe_xCr_{1-x}S$ solid solutions. *Phys. Solid State* **49**, 1920-1922 (2007).
3. Néel, L. Magnetic properties of ferrites: Ferrimagnetism and antiferromagnetism. *Ann. Phys.* **12**, 137-198 (1948).
4. Néel, L. Magnetism and local molecular field. *Science* **174**, 985-992 (1971).
